# Differential Response of Floating and Submerged Leaves of Longleaf Pondweed to Silver Ions

**DOI:** 10.3389/fpls.2017.01052

**Published:** 2017-06-21

**Authors:** Nisha Shabnam, P. Sharmila, Govindjee, Hyunook Kim, P. Pardha-Saradhi

**Affiliations:** ^1^Department of Energy and Environmental System Engineering, University of SeoulSeoul, South Korea; ^2^Department of Chemistry, Indian Institute of Technology DelhiNew Delhi, India; ^3^Center for Biophysics and Quantitative Biology, University of Illinois at Urbana-Champaign, UrbanaIL, United States; ^4^Department of Environmental Studies, University of DelhiNew Delhi, India

**Keywords:** antioxidants, Ag-nanoparticles, ecophysiological adaptation, heterophyllous aquatic plant, photosystem II, *Potamogeton nodosus*

## Abstract

In this study, we have investigated variations in the potential of floating and submerged leaves of longleaf pondweed (*Potamogeton nodosus*) to withstand silver ion (Ag^+^)-toxicity. Both floating and submerged leaves changed clear colorless AgNO_3_ solutions to colloidal brown in the presence of light. Transmission electron microscopy revealed the presence of distinct crystalline Ag-nanoparticles (Ag-NPs) in these brown solutions. Powder X-ray diffraction pattern showed that Ag-NPs were composed of Ag^0^ and Ag_2_O. Photosystem (PS) II efficiency of leaves declined upon exposure to Ag^+^ with a significantly higher decline in the submerged leaves than in the floating leaves. Similarly, Ag^+^ treatment caused a significant reduction in the carboxylase activity of the ribulose bisphosphate carboxylase/oxygenase in leaves. The reduction in this carboxylase activity was significantly higher in the submerged than in the floating leaves. Ag^+^ treatment also resulted in a significant decline in the levels of non-enzymatic and enzymatic antioxidants; the decline was significantly lower in the floating than in submerged leaves. X-ray photoelectron spectroscopy revealed the presence of Ag_2_O in these leaves. Inductively coupled plasma mass spectrometry analysis revealed a three-fold higher Ag content in the submerged than in floating leaves. Our study demonstrates that floating leaves of longleaf pondweed have a superior potential to counter Ag^+^-toxicity compared with submerged leaves, which could be due to superior potential of floating leaves to reduce Ag^+^ to less/non-toxic Ag^0^/Ag_2_O-nanoparticles/nanocomplexes. We suggest that modulating the genotype of longleaf pondweed to bear higher proportion of floating leaves would help in cleaning fresh water bodies contaminated with ionic forms of heavy metals.

## Introduction

Human activities, in particular, industrialization and urbanization, have led to a drastic acceleration in heavy metal pollution of our surroundings and ecosystems (Nriagu, 1996). Negative impact of heavy metal(s) on the health of living beings (including the humans) and ecosystems is of serious concern; this effect is being increasingly felt over the past few decades. In view of its ecofriendly nature, bioremediation (i.e., use of living beings or their components for detoxification of pollutants through, e.g., transformation, and degradation) is being used as one of the key approaches to decrease the level of heavy metals in the surroundings (Dhir et al., 2009; Rai, 2009). Microbial-assisted removal of heavy metals has been a popular bioremediation process. However, due to difficulties in harnessing the microbes from soils or water, plant-based bio-sorption of heavy metals is now receiving greater attention across the world. A large number of plants are hyper-accumulators of heavy metals, so many researchers are now trying to understand hyper-accumulating strategies in these plants (Kamal et al., 2004; Dhir et al., 2009; Sharma and Dietz, 2009). Pardha-Saradhi et al. (2014a,b,c) have shown that terrestrial plants have the potential to biotransform precious heavy metal ions (e.g., Au^3+^ and Ag^+^) and essential metal ions (e.g., Fe^3+^) into less/non-toxic nanoparticles (NPs)/nanocomplexes. Shabnam et al. (2017) have recently demonstrated that Ag-NPs are significantly less toxic than ionic Ag.

Heavy metals released from industries and other sources often find their way into water bodies, e.g., lakes, rivers, and oceans (Rai, 2009). Phytoplanktons contribute to over 50% of the organic material produced through photosynthetic CO_2_ fixation (Arrigo, 2005). However, research has, thus far, been focused mainly on the macrophytes simply because of the ease with which they can be handled and harvested. Amongst the macrophytes, attention has been given mostly to homophyllous aquatic plants. In spite of being better adapted to the fluctuating climatic conditions compared to homophyllous aquatic macrophytes, heterophyllous aquatic plants have received less attention from the researchers (Iida et al., 2009).

Pardha-Saradhi et al. (2014a) have used silver as an ideal model heavy metal, since response of plants to Ag^+^ can be visually recorded and easily characterized. Anthropogenic activities such as mining, electroplating and photographic industry are responsible for the release of silver into our surroundings (Purcell and Peters, 1998; Ratte, 1999). No attempt has, thus far, been made to evaluate the impact of silver on any heterophyllous aquatic macrophyte. Longleaf pondweed has floating and submerged leaves. While floating leaves are present on the surface, submerged leaves are under water. Previously, we reported that the floating leaves have superior photosynthetic efficiency and antioxidant system compared to the submerged ones (Shabnam et al., 2015; Shabnam and Pardha-Saradhi, 2016). Therefore, in this study, we chose this plant to evaluate differences in the tolerance of these types of leaves to Ag^+^ toxicity. We have evaluated the impact of silver on photosynthesis and antioxidant system. Our findings revealed that these leaves possess potential to generate Ag-NPs on exposure to Ag^+^. We believe that this potential of leaves to generate Ag-NPs is a mechanism to restrict the uptake of Ag^+^ and thus, counter its toxic effects.

## Materials and Methods

### Experimental Procedure

Longleaf pondweed (*Potamogeton nodosus*, Potamogetonaceae) was grown at the University of Delhi, as described by Shabnam et al. (2015). For studying the impact of Ag^+^ on floating and submerged leaves of longleaf pondweed, fully expanded mature leaves were used. Leaves, after washing three times with double-distilled water, were acclimatized under laboratory conditions for 3 h. Silver nitrate (AgNO_3_) was used to impose silver ion (Ag^+^)-toxicity. The leaves were exposed to different levels (0, 5, 10, 50, 100, 250, and 500 μM) of AgNO_3_ in Borosil dishes (190 mm diameter × 100 mm height) under continuous white light (120 μmol photons m^-2^ s^-1^) for 24 h. Understandably, in this experimental setup floating leaves float and submerged leaves get submerged in test solution during experimental exposure.

Impact of Ag^+^ on floating and submerged leaves of longleaf pondweed was evaluated by measuring (i) photosystem (PS) II efficiency; (ii) carboxylase activity of ribulose bisphosphate carboxylase/oxygenase (Rubisco); and (iii) enzymatic and non-enzymatic antioxidants according to the protocols of Shabnam and Pardha-Saradhi (2016).

### Analytical Methods

#### Photosystem II Efficiency

For determining PS II efficiency, leaves were dark-adapted for 40 min and Chl *a* fluorescence induction measurements were made on 8–10 different portions of leaves. Fluorescence transient, from 10 μs to 1 s, was measured using a plant efficiency analyzer (PEA) (Handy PEA; Hansatech Ltd, Norfolk, United Kingdom); leaves were excited with red light (peak at 650 nm) at an intensity of 3,500 μmol photons m^-2^ s^-1^, provided by an array of six light-emitting diodes (LEDs). At least five leaves were used for each treatment. Biolyzer software HP 3 (Bioenergetics Laboratory, University of Geneva, Geneva, Switzerland) was used to plot Chl *a* fluorescence data. For details on measurement of Chl *a* fluorescence, see Shabnam et al. (2015). Quantum efficiency of PS II activity was inferred from the ratio of variable (*F*_v_) to maximum (*F*_m_) Chl *a* fluorescence, where *F*_v_ = *F*_m_ -*F*_o_, *F*_o_ being the minimum fluorescence (see Govindjee, 2004). Chl *a* and Chl *b* levels of leaves were quantified according to the method and equations used by Arnon (1949).

#### Carboxylase Activity of Rubisco

Carboxylase activity of Rubisco (EC 4.1.1.39) was measured as described earlier (Shabnam and Pardha-Saradhi, 2016). Leaves were homogenized in chilled 50 mM Tris–HCl buffer (pH 7.6) containing 1 mM DTT, 5 mM EDTA and 5% PVP with acid washed sand in pre-chilled mortar and pestle. The homogenate was centrifuged at 15,000 × *g* for 20 min at 4°C, and the supernatant was used as a crude enzyme. The carboxylase activity of Rubisco was measured, at 25 ± 2°C, using an assay mixture containing crude enzyme extract, Tris-HCl buffer (200 mM, pH 8.5), 1 mM RuBP, 10 mM NaHCO_3_, 5 mM MgCl_2_, 0.1 mM DTT, 1 mM ATP, 5 units of phosphoglycerate kinase, 5 units of glyceraldehyde-3-phosphate dehydrogenase, and 0.2 mM NADH. Oxidation of NADH was recorded as decrease in absorbance at 340 nm; the carboxylase activity of Rubisco was initially calculated in terms of nmoles of NADH oxidized min^-1^ g^-1^ fresh weight. Subsequently carboxylase activity of Rubisco was extrapolated and expressed in terms of CO_2_ fixed min^-1^ g^-1^ fresh weight.

#### Determination of Silver in Leaves

Silver content of leaves was measured using inductively coupled plasma mass spectrometry (ICP-MS) (NexION 300D, Perkin Elmer, Waltham, MA, United States) and expressed as mg silver g^-1^ dry weight. Leaves exposed to Ag^+^ were also analyzed by X-ray photoelectron spectroscopy (XPS; Phi 5000 VersaProbe, Ulvac-Phi, Chigasaki, Japan).

#### Determination of Non-enzymatic Antioxidants

Levels of non-enzymatic antioxidants (i.e., phenolics, thiols, and ascorbate) were measured as described earlier (Shabnam and Pardha-Saradhi, 2016). Leaves were homogenized in chilled 5% TCA with mortar and pestle. The homogenate was centrifuged at 20,000 × *g* for 15 min at 4°C, and the supernatant was used for determining the levels of total ascorbate, total phenolics and thiols, as described below.

##### Total ascorbate

The reaction mixture consisted of 200 μl supernatant, 100 μl DTT (10 mM), 100 μl NEM (0.5%), 500 μl TCA (10%), 400 μl orthophosphoric acid (43%), 400 μl α-α′-bipyridyl (4%) and 200 μl FeCl_3_ (3%); it was immediately vortexed to avoid the formation of any precipitate. This reaction mixture was incubated at 37°C for 1 h and the absorbance was measured at 525 nm. The amount of total ascorbate was expressed as nmoles g^-1^ fresh weight.

##### Total phenolics

One ml of supernatant was incubated with a mixture of 1 ml Folin–Ciocalteu reagent and 2 ml Na_2_CO_3_ (700 mM) for 1 h in dark at room temperature. Subsequently, absorbance of the reaction mixture was measured at 765 nm. Total phenolic content was expressed as nmoles of GAE g^-1^ fresh weight, using a standard curve obtained with gallic acid.

##### Thiols

To 200 μl supernatant, 775 μl K_2_HPO_4_ (500 mM) and 25 μl 5,5′-Dithiobis (2-nitrobenzoic acid) (DTNB) (10 mM in 100 mM phosphate buffer, pH 7.0) were added. Absorbance of the samples was measured at 412 nm and corrected against the absorbance of a sample without added DTNB. Thiol content was expressed as nmoles g^-1^ fresh weight, using an extinction coefficient of 13.6 mM^-1^ cm^-1^ at 412 nm.

#### Determination of Activities of Enzymatic Antioxidants

The activities of antioxidant enzymes, such as SOD (EC 1.15.1.1), catalase (EC 1.11.1.6), GPX (EC 1.11.1.7), ascorbate peroxidase (APX, EC 1.11.1.11), MDHAR (EC 1.6.5.4), DHA reductase (DHAR, EC 1.8.5.1), and GR (EC 1.6.4.2) were measured as described earlier (Shabnam and Pardha-Saradhi, 2016). Leaves were homogenized in chilled 50 mM Tris–HCl buffer (pH 7.6) containing 1 mM DTT, 5 mM EDTA and 5% PVP with acid washed sand in chilled mortar and pestle. The homogenate was centrifuged at 15,000 × *g* for 20 min at 4°C. The supernatant was taken as a crude enzyme extract and was used for estimating activities of various antioxidant enzymes as briefly described below.

##### Superoxide dismutase

To 4 ml of 200 mM Tris-HCl buffer (pH 7.6), we added 200 μl L-methionine (20 mM), 200 μl EDTA (0.1 mM), 100 μl hydroxylamine, 100 μl Triton X (0.1%), 200 μl riboflavin (0.5 mM) and the enzyme extract. Tubes containing the resultant reaction mixture were exposed to 120 μmol photons m^-2^ s^-1^ of white light, using an incandescent lamp, at 25 ± 2°C. After exposure to light for 45 min, 2 ml of freshly prepared Greiss reagent [containing equal volumes of 0.1% naphthylethylenediamine dihydrochloride (NED) and 1% sulphanilamide dissolved in 5% orthophosphoric acid] was added to the reaction mixture and absorbance was measured at 543 nm. SOD activity was expressed in terms of nmoles of O_2_^-^ consumed min^-1^ g^-1^ fresh weight.

##### Catalase

Activity of catalase (CAT, EC 1.11.1.6) was determined by measuring the rate of oxygen evolution in a reaction mixture containing the enzyme extract in 200 mM phosphate buffer (pH 6.5) and 20 mM H_2_O_2_ at 25 ± 2°C, using a Clark-type liquid phase O_2_ electrode (Hansatech, United Kingdom). The enzyme activity was expressed as nmoles of oxygen evolved min^-1^ g^-1^ fresh weight.

##### Guaiacol peroxidase

The reaction mixture for determining activity of guaiacol peroxidase activity consisted of a reaction mixture consisting of 200 mM phosphate buffer (pH 6.5), 2 mM guaiacol and 20 mM H_2_O_2_ incubated with enzyme extract, at 25 ± 2°C. Enzyme activity was measured by recording increase in absorbance at 470 nm with time. The enzyme activity was expressed as nmoles of tetraguaiacol formed min^-1^ g^-1^ fresh weight, using an extinction coefficient of 26.6 mM^-1^ cm^-1^ at 470 nm.

##### Ascorbate peroxidase

Ascorbate peroxidase (APX, EC 1.11.1.11) activity was determined by estimating the rate of oxidation of ascorbate at 290 nm in a reaction mixture consisting of 200 mM Tris-HCl buffer (pH 7.6), 20 mM H_2_O_2_, 1 mM sodium azide, 2 mM ascorbate and the enzyme extract, at 25 ± 2°C. The activity of APX was expressed as nmoles of ascorbate oxidized min^-1^ g^-1^ fresh weight, using an extinction coefficient of 2.8 mM^-1^ cm^-1^, at 290 nm.

##### Monodehydroascorbate reductase

For measuring activity of MDHAR (EC 1.6.5.4), the reaction mixture consisted of 200 mM Tris-HCl buffer (pH 7.6), the enzyme extract, 2 mM ascorbate, 10 units of ascorbate oxidase and 0.2 mM NADH. Decrease in absorbance at 340 nm, due to the oxidation of NADH, was measured at 25 ± 2°C. Extinction coefficient of 6.2 mM^-1^ cm^-1^ (at 340 nm) was used to express the activity of MDHAR as nmoles of NADH oxidized min^-1^ g^-1^ fresh weight.

##### Dehydroascorbate reductase

The reaction mixture for the determination of dehydroascorbate reductase (DHAR, EC 1.8.5.1) activity included 200 mM Tris-HCl buffer (pH 7.6), enzyme extract, 1 mM reduced glutathione (GSH), and 1 mM dehydroascorbate. An increase in absorbance at 265 nm, due to the formation of ascorbate, from DHA by DHAR, in the presence of GSH, was measured at 25 ± 2°C. Enzyme activity was expressed in terms of nmoles of ascorbate formed min^-1^ g^-1^ fresh weight, using an extinction coefficient of 14 mM^-1^ cm^-1^at 265 nm.

##### Glutathione reductase

The reaction mixture for measuring GR (EC 1.6.4.2) activity consisted of 200 mM Tris-HCl buffer (pH 7.6), enzyme extract, 1 mM oxidized glutathione (GSSG) and 0.2 mM NADH. Decrease in absorbance at 340 nm, due to the oxidation of NADH, was measured at 25 ± 2°C. The activity of GR was expressed as nmoles of NADH oxidized min^-1^ g^-1^ fresh weight, using extinction coefficient of 6.2 mM^-1^ cm^-1^ (at 340 nm).

#### Characterization of Ag-NPs

For TEM studies, 10 μl of colloidal solution was drop-coated on a 200 mesh copper grid with an ultrathin continuous carbon film, and allowed to dry in a desiccator at room temperature. Grids were viewed under a TEM (Technai G2 T30, Lonate Pozzolo, Italy) at a voltage of 300 KV. The hardware associated with the instrument allowed us to obtain (i) the EDX analysis to measure the elemental composition of the particle sample; and (ii) the SAED analysis to determine the crystalline/amorphous nature of NPs.

For PXRD studies, colloidal solutions were centrifuged. The pellet obtained was re-suspended in distilled water, drop-coated on silica surface, dried in a desiccator, and then used for collecting PXRD pattern, using Rigaku Rotaflex RAD-B with copper target CuK(α)1 radiation, with a tube voltage of 40 kV and a current of 60 mA in 2 theta (𝜃) range of 30–80°.

### Statistical Analysis

All the experiments were carried out independently six times. The data obtained were statistically tested with ANOVA using the general linear model. The variations between the means of treatments were compared using Duncan’s multiple range test (at *P* ≤ 0.05). All these statistical analyses were performed using IBM-SPSS statistical software, version 22.0 (IBM Corporation, Armonk, NY, United States).

## Results

### Potential of Floating and Submerged Leaves to Generate Ag-NPs

**FIGURE 1:**
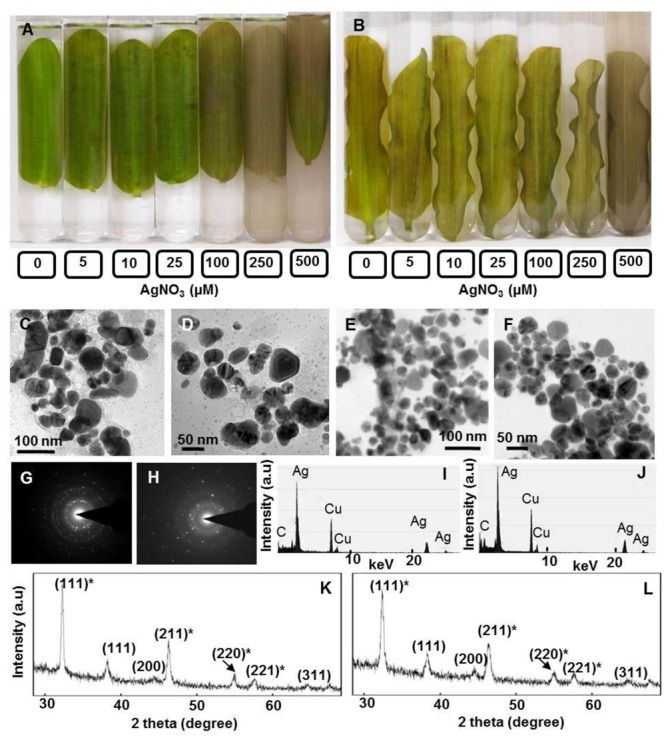
Potential of floating and submerged leaves of longleaf pondweed (*Potamogeton nodosus*) to generate Ag-NPs. Photographs were taken after 24 h exposure of floating **(A)** and submerged **(B)** leaves to varying concentrations of AgNO_3_ (in μM) in Borosil dishes (190 mm diameter × 100 mm height, in order to ensure that floating leaves remain floating and submerged leaves remain submerged in respective test solution during the course of exposure to AgNO_3_). TEM **(C–F)**, SAED pattern **(G,H)**, EDX **(I,J)** and PXRD patterns **(K,L)** of Ag^0^/Ag_2_O-NPs generated by floating **(C,D,G,I)** and submerged leaves **(E,F,J,L)** of longleaf pondweed. PXRD patterns **(K,L)** show Bragg reflections specific to crystalline face-centered cubic structure of Ag^0^ (in parenthesis without star) and cubic structure of Ag_2_O (in parenthesis with star).

We observed alteration of clear colorless AgNO_3_ solutions to colloidal brown when incubated with floating and submerged leaves of longleaf pondweed within 24 h (**Figures 1A,B**). Clear colorless AgNO_3_ solutions turned colloidal brown due to the formation of Ag-NPs (Shabnam et al., 2016). AgNO_3_ solutions incubated in the absence of leaves did not show any alteration in color, thus confirming that leaves were responsible for the observed color change. Floating leaves turned AgNO_3_ solutions colloidal brown more intensively compared to the submerged leaves, although only one side of floating leaves was in contact with test solution. Supplementary Figure 1 shows experimental setup revealing that the floating leaves possess superior potential to turn clear colorless AgNO_3_ (500 μM) solutions colloidal brown compared to submerged leaves. For depicting the gradation in color with better clarity the test solutions (i.e., different concentrations of AgNO_3_) incubated with floating and submerged leaves for 24 h were transferred to test tubes along with leaves (**Figures 1A,B**). However, absorption spectra of the brown colloidal solution did not show any Ag-NP specific absorption peak.

Transmission electron microscopy revealed the presence of distinct NPs of varying shapes and sizes (∼10–80 nm) in these colloidal brown solutions (**Figures 1C–F**). EDX of these NPs showed peaks specific to Ag (**Figures 1I,J**). SAED pattern revealed the crystalline nature of these Ag-NPs (**Figures 1G,H**). PXRD patterns showed Bragg reflections (111), (200), and (311), revealing crystalline nature and face centered cubic structure of Ag^0^-NPs (**Figures 1K,L**) (Pardha-Saradhi et al., 2014a). Additional peaks observed in the PXRD spectra might be due to Bragg reflections (111)^∗^, (211)^∗^, (220)^∗^, (221)^∗^ of cubic Ag_2_O (**Figures 1K,L**).

### Impact of Ag^+^ on Photosynthesis in Floating and Submerged Leaves

**FIGURE 2:**
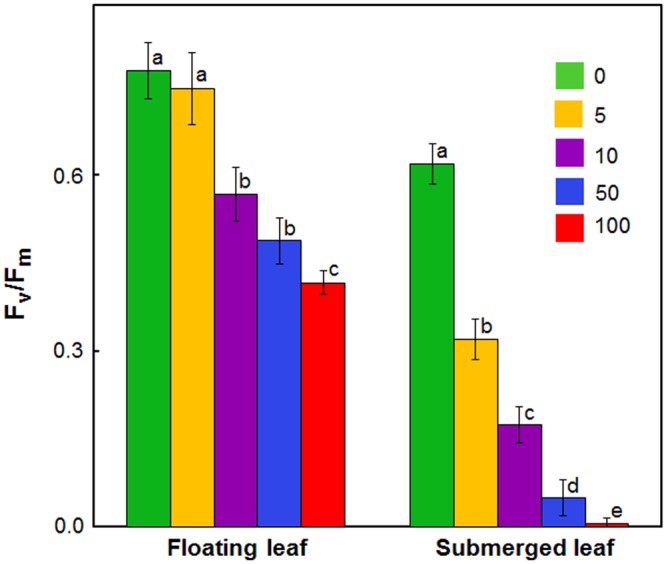
Impact of Ag^+^ on the quantum yield of Photosystem II activity (as inferred from *F*_v_/*F*_m_; see text) of floating and submerged leaves of longleaf pondweed (*P. nodosus*) after 24 h exposure to varying concentrations of AgNO_3_ (μM). Data are a mean of recordings from six independent experiments. Vertical lines on data points represent standard errors. Different small letters over the bars reflects that the values recorded for leaves (floating or submerged) exposed to different concentrations of AgNO_3_ do not differ significantly at *P* ≤ 0.05 level (Duncan’s multiple range test).

In view of large differences in photosynthetic activities between floating and submerged leaves (Shabnam et al., 2015), we examined the effects of Ag^+^ on Photosystem II (PS II) efficiency of these leaves. Photosystem II efficiency (maximum quantum yield) is often determined as a ratio of variable Chl *a* fluorescence to maximum Chl *a*, i.e., *F*_v_/*F*_m_. Chl *a* fluorescence of oxygenic organisms shows a rise from a basal level (*F*_o_) (i.e., minimum fluorescence) to the maximum (*F*_m_) (Strasser et al., 1995; Stirbet and Govindjee, 2012; Shabnam et al., 2015, 2017). *F*_o_ and *F*_m_ values of both floating and submerged leaves declined significantly on exposure to Ag^+^; the decline was significantly higher in the submerged leaves than in the floating ones (**Table 1**). Ag^+^, like other heavy metal ions, brought about a significant decline in the quantum yield of PS II activity, as inferred from *F*_v_/*F*_m_ values, in both floating and submerged leaves (**Figure 2**). However, at any given concentration, the decline in *F*_v_/*F*_m_ was significantly higher in the submerged leaves.

**Table 1:** Variations in *F*_o_ (the minimum fluorescence) and *F*_m_ (maximum fluorescence) of floating and submerged leaves of longleaf pondweed (*Potamogeton nodosus*) exposed to different concentrations of AgNO_3_.

Ag^+^ (μM)	*F*_o_		*F*_m_	
	Floating	Submerged	Floating	Submerged
0	302 ± 29.6^a^ (100)	468 ± 26.1^a^ (100)	1355 ± 87.7^a^ (100)	1272 ± 101.8^a^ (100)
5	255 ± 18.5^a^ (84.4)	257 ± 24.3^b^ (54.9)	936 ± 68.3^b^ (69.1)	377 ± 21.1^b^ (29.6)
10	238 ± 21.6^ab^ (78.8)	253 ± 19.7^b^ (54.1)	549 ± 39.9^c^ (40.5)	391 ± 19.8^b^ (30.7)
50	257 ± 11.7^a^ (85.1)	250 ± 17.5^b^ (53.4)	501 ± 42.4^c^ (36.9)	299 ± 23.7^c^ (23.5)
100	213 ± 13.3^b^ (70.5)	248 ± 11.8^b^ (52.9)	365 ± 24.5^d^ (26.9)	311 ± 28.5^c^ (24.4)
250	162 ± 11.3^c^ (53.6)	258 ± 26.7^b^ (55.1)	160 ± 11.1^e^ (11.8)	245 ± 16.9^d^ (19.3)
500	152 ± 11.1^c^ (50.3)	142 ± 11.5^c^ (30.3)	144 ± 8.9^e^ (10.6)	128 ± 9.6^e^ (10.1)

*Data are a mean of recordings from six independent experiments. Data represent mean ± standard error. Values followed by the same small letter (in superscript) within a column do not differ significantly at *P* ≤ 0.05 level (Duncan’s multiple range test). Values in parenthesis represent the percent change over respective controls.*

**FIGURE 3:**
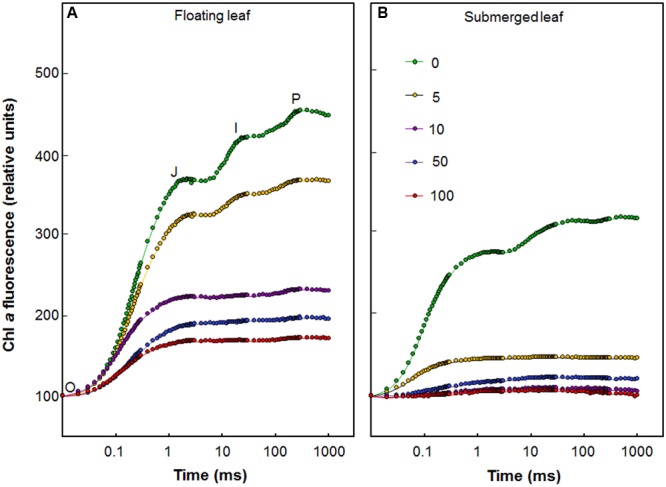
Effect of Ag^+^ on chlorophyll *a* fluorescence transient, the OJIP curve (see text), of floating and submerged leaves of longleaf pondweed (*P. nodosus*) after 24 h exposure to varying concentrations of AgNO_3_ (in μM). Chl *a* fluorescence induction curves of floating **(A)** and submerged **(B)** leaves of longleaf pondweed exposed to varying concentrations of AgNO_3_ (in μM) for 24 h. Chl *a* fluorescence induction curves were plotted by normalizing data at the *F*_o_ values.

**Figure 3** shows the fast (up to a second) polyphasic Chl *a* fluorescence transients of floating and submerged leaves which were exposed to different Ag^+^ levels. All oxygen-evolving organisms show polyphasic Chl *a* fluorescence transients (also called the OJIP curves) with distinct O, J, I, and P steps (**Figure 3**). In these curves, “O” is the minimum fluorescence (*F*_o_), “P” is the peak (*F*_m_), and “J” and “I” are intermediate levels. The polyphasic rise of Chl *a* fluorescence transient was severely reduced in both floating and submerged leaves, exposed to Ag^+^ (**Figure 3**), which is in agreement with the decline in *F*_v_/*F*_m_. The decline in the amplitude of fluorescence was significantly higher in submerged leaves than in floating leaves, revealing superior potential of the latter to tolerate Ag^+^ than the former. Extreme sensitivity of submerged leaves to Ag^+^-toxicity was also evident from the loss in the polyphasic nature of Chl *a* fluorescence transients even at concentration as low as 5 μM of Ag^+^.

Chlorophyll *a* fluorescence kinetics is affected by such factors as Chl content. Therefore, we evaluated the impact of 24 h Ag^+^ treatment on both Chl *a* and Chl *b* levels. Decline of these Chls was recorded in both floating and submerged leaves, as a function of silver concentration (**Table 2**). However, the decline in the levels of both Chl *a* and Chl *b* levels were significantly lower in the floating leaves. Interestingly, decline in levels of Chl *a* was significantly higher than that of Chl *b* in both floating and submerged leaves.

**Table 2:** Variation in Chl *a* and Chl *b* levels (μg g^-1^ fresh weight) in floating and submerged leaves of longleaf pondweed (*P. nodosus*) exposed to different concentrations of AgNO_3_.

Ag^+^ (μM)	Chl *a*		Chl *b*	
	Floating	Submerged	Floating	Submerged
0	626 ± 33.3^a^ (100)	490 ± 27.3^a^ (100)	492 ± 21.5^a^ (100)	471 ± 20.1^a^ (100)
5	598 ± 28.9^a^ (95.5)	438 ± 30.3^a^ (89.4)	486 ± 26.7^a^ (98.8)	429 ± 25.4^a^ (91.1)
10	492 ± 31.1^b^ (78.6)	368 ± 23.7^c^ (75.1)	391 ± 23.3^b^ (79.4)	367 ± 26.7^b^ (77.9)
50	431 ± 23.3^b^ (68.8)	298 ± 16.9^d^ (60.8)	381 ± 18.4^b^ (77.4)	342 ± 19.5^bc^ (72.6)
100	401 ± 23.5^bc^ (64.1)	214 ± 21.1^e^ (43.7)	368 ± 25.4^bc^ (74.7)	273 ± 20.3^c^ (57.9)
250	369 ± 19.7^c^ (58.9)	184 ± 14.5^e^ (37.6)	340 ± 16.7^c^ (69.1)	242 ± 14.7^c^ (51.3)
500	227 ± 20.8^d^ (36.2)	144 ± 9.1^f^ (29.4)	298 ± 17.4^d^ (60.6)	191 ± 16.7^d^ (40.5)

*Data are a mean of recordings from six independent experiments. Data represent mean ± standard error. Values followed by same small letter (in superscript) within a column do not differ significantly at *P* ≤ 0.05 level (Duncan’s multiple range test). Values in parenthesis represent the percent change over respective controls.*

**FIGURE 4:**
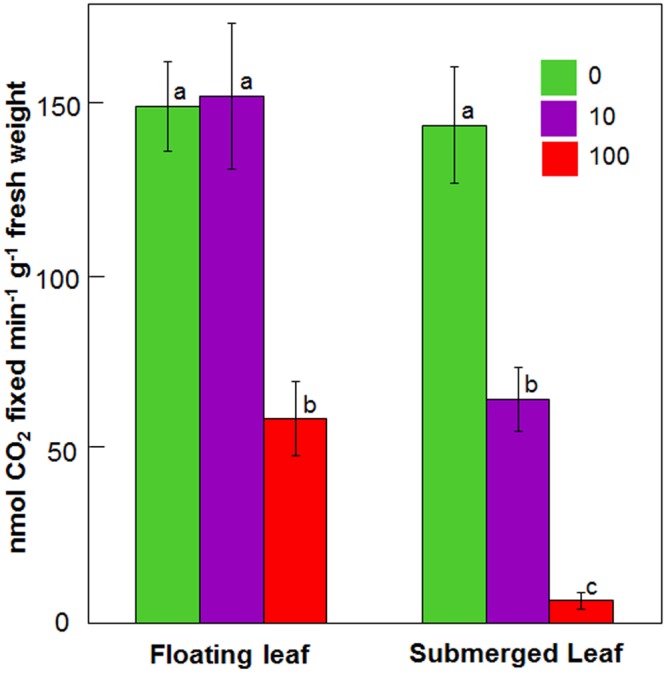
Impact of Ag^+^ on the carboxylase activity of Rubisco of floating and submerged leaves of longleaf pondweed (*P. nodosus*) after 24 h exposure to 10 or 100 μM AgNO_3_. Data are a mean of recordings from six independent experiments. Vertical lines on bars represent standard errors. Different small letters over the bars reflect that the values recorded for leaves (floating or submerged) exposed to different concentrations of AgNO_3_ do not differ significantly at *P* ≤ 0.05 level (Duncan’s multiple range test).

**Figure 4** depicts impact of 24 h Ag^+^ treatment on carboxylase activity of Rubisco in floating and submerged leaves. Upon exposure to 10 μM Ag^+^, the carboxylase activity of Rubisco declined by 50% in the submerged leaves, whereas it remained unaltered in the floating ones. However, 100 μM Ag^+^ caused ∼50 and ∼90% decline in Rubisco activity in the floating and submerged leaves, respectively (**Figure 4**).

### Levels of Ag in Floating and Submerged Leaves

**FIGURE 5:**
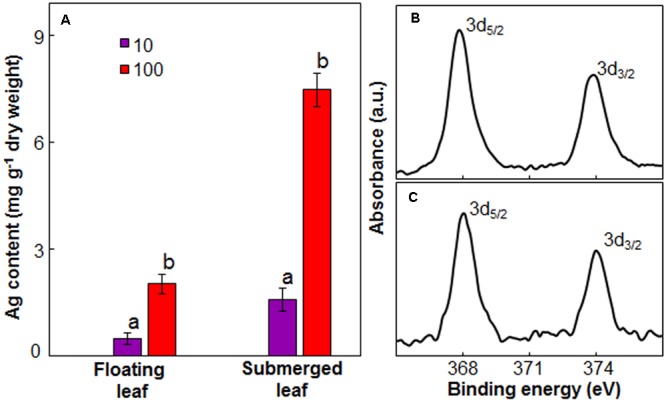
Ag content in floating and submerged leaves of longleaf pondweed (*P. nodosus*) after 24 h exposure to 10 or 100 μM AgNO_3_. **(A)** Bars are a mean of data from three independent experiments for different treatments. Vertical lines on data points represent standard errors. Different small letters over the bars reflect that the values recorded for leaves (floating or submerged) exposed to different concentrations of AgNO_3_ do not differ significantly at *P* ≤ 0.05 level (Duncan’s multiple range test). High resolution XPS **(B,C)** of floating **(B)** and submerged **(C)** leaves showing presence of peaks specific to Ag_2_O.

Since there was a significant variation in the impact of Ag^+^ on photosynthetic efficiency between floating and submerged leaves, we measured Ag content in these leaves. Both floating and submerged leaves of longleaf pondweed exposed to Ag^+^ showed the presence of Ag. The level of Ag in these leaves increased as a function of Ag^+^ concentration to which they were exposed (**Figure 5A**). At any Ag^+^ concentration, silver content in submerged leaves was ∼3 times higher than that in floating ones. XPS analysis also confirmed the presence of Ag in both leaves. XPS spectra showed two peaks at binding energies of 368 and 374 eV (**Figures 5B,C**), which arise due to the emission of 3d_5/2_ and 3d_3/2_ photoelectrons, respectively (Adegboyega et al., 2013).

### Impact of Ag^+^ on Antioxidant System in Floating and Submerged Leaves

**FIGURE 6:**
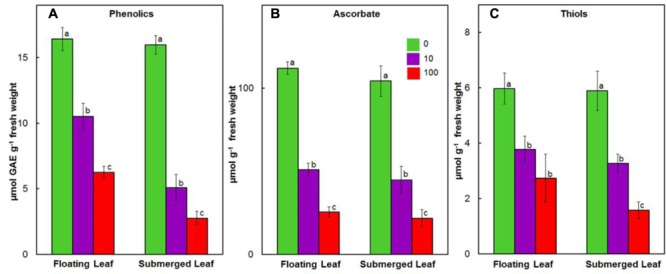
Impact of Ag^+^ on the levels of non-enzymatic antioxidants. Levels of phenolics **(A)**; ascorbate **(B)**; and thiols **(C)** in floating and submerged leaves of longleaf pondweed (*P. nodosus*) after 24 h exposure to 10 or 100 μM AgNO_3_. Bars are a mean of data from six independent experiments for different treatments. Vertical lines on bars represent standard errors. Different small letters over the bars reflect that the values recorded for leaves (floating or submerged) exposed to different concentrations of AgNO_3_ do not differ significantly at *P* ≤ 0.05 level (Duncan’s multiple range test).

Like our earlier findings (Shabnam and Pardha-Saradhi, 2016), we did not observe any significant variation in the levels of ascorbate, phenolics, and thiols amongst floating and submerged leaves which were not exposed to Ag^+^. However, both floating and submerged leaves exposed to 10 and 100 μM Ag^+^ showed a decline in the levels of all these three non-enzymatic antioxidants (**Figure 6**). Irrespective of the Ag^+^ concentrations to which leaves were exposed, the decline in the level of phenolics was significantly higher in the submerged leaves (**Figure 6A**). However, the decline in the levels of ascorbate and thiols was almost similar for both leaves (**Figures 6B,C**).

**FIGURE 7:**
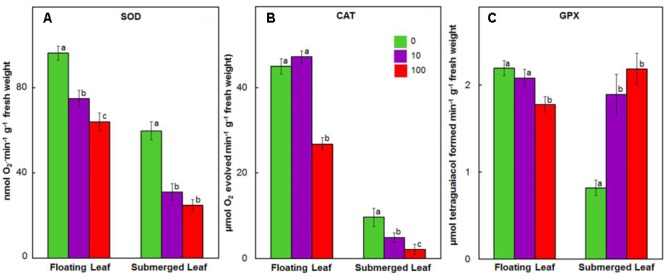
Impact of Ag^+^ on the activities of antioxidant enzymes. Activity of SOD **(A)**; catalase (CAT) **(B)**, and guaiacol peroxidase (GPX) **(C)** in floating and submerged leaves of longleaf pondweed (*P. nodosus*) after 24 h exposure to 10 or 100 μM AgNO_3_. Bars are a mean of data from six independent experiments for different treatments. Vertical lines on data points represent standard errors. Different small letters over the bars reflect that the values recorded for leaves (floating or submerged) exposed to different concentrations of AgNO_3_ do not differ significantly at *P* ≤ 0.05 level (Duncan’s multiple range test).

Antioxidant enzymes, such as SOD, catalase (CAT), GPX, ascorbate peroxidase (APX), MDHAR, dehydroascorbate reductase (DHAR), and GR play an important role in scavenging ROS in plants exposed to heavy metals (Prasad et al., 1999; Dhir et al., 2009). Therefore, we evaluated the impact of Ag^+^ on activities of these enzymes in both floating and submerged leaves of longleaf pondweed. Ag^+^ treatment caused a significant decline in SOD activity in both floating and submerged leaves, although the decline was significantly higher in the latter compared to the former (**Figure 7A**). Both floating and submerged leaves, with the exception of floating leaves exposed to 10 μM Ag^+^, showed a significant decrease in the catalase activity compared to their respective controls (**Figure 7B**). However, the degree of loss in catalase activity was higher in submerged leaves. Contrary to decreased activity of SOD and catalase, activity of GPX increased by 2–2.5-fold in submerged leaves exposed to Ag^+^ (**Figure 7C**). However, floating leaves showed a decrease in GPX activity on exposure to Ag^+^.

**FIGURE 8:**
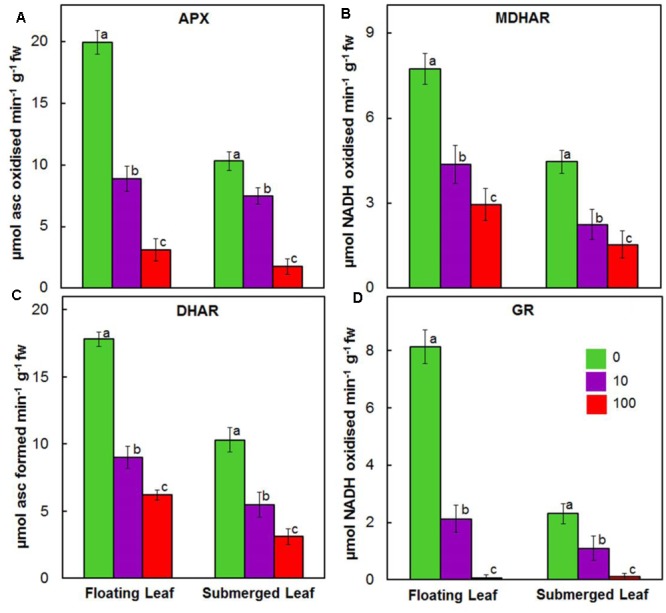
Impact of Ag^+^ on the activities of antioxidant enzymes of ascorbate-glutathione cycle. Activity of ascorbate peroxidase (APX) **(A)**; monodehydroascorbate reductase (MDHAR) **(B)**; dehydroascorbate reductase (DHAR) **(C)**; and glutathione reductase (GR) **(D)** in floating and submerged leaves of longleaf pondweed (*P. nodosus*) after 24 h exposure to 10 or 100 μM AgNO_3_. Data are a mean of recordings from six independent experiments. Vertical lines on data points represent standard errors. Different small letter over the bars reflects that the values recorded for leaves (floating or submerged) exposed to different concentrations of AgNO_3_ do not differ significantly at *P* ≤ 0.05 level (Duncan’s multiple range test).

Activities of enzymes of ascorbate-glutathione cycle, e.g., APX, MDHAR, DHAR, and GR, decreased significantly in both floating and submerged leaves as a function of given Ag^+^ level (**Figure 8**). In general, irrespective of the concentration of Ag^+^ used, the activities of ascorbate-glutathione cycle enzymes were significantly higher in floating leaves. Amongst enzymes of ascorbate-glutathione cycle, Ag^+^ induced highest decrease in the activity of GR, followed by those of APX, MDHAR, and DHAR.

## Discussion

### Leaves of Longleaf Pondweed Generate Ag-NPs as a Defense Mechanism

Earlier, Pardha-Saradhi et al. (2014a,b,c) reported that plants reduce toxic ionic forms of heavy metals into non/less-toxic NPs as a defense mechanism. During our investigations, we found that both floating and submerged leaves of longleaf pondweed could turn clear colorless AgNO_3_ solutions to colloidal brown (**Figures 1A,B** and Supplementary Figure 1). TEM coupled with SAED and EDX confirmed the presence of distinct crystalline NPs composed of Ag in these colloidal brown solutions (**Figures 1C–F**). However, similar to our earlier findings (Pardha-Saradhi et al., 2014a), these brown colloidal solutions did not show any Ag-NP specific peak in the absorption spectra. In addition to SAED, PXRD confirmed the crystalline nature of Ag-NPs; PXRD pattern showed peaks specific to face centered cubic structured Ag^0^ and cubic structured Ag_2_O. These PXRD analyses clearly showed that Ag-NPs generated by floating and submerged leaves are composed of both Ag^0^ and Ag_2_O. XPS analysis confirmed the presence of Ag in both leaves and that the accumulated Ag in these leaves existed predominantly as Ag_2_O state. It is well-known that Ag^0^ and Ag^0^-NPs are prone to oxidation (Pardha-Saradhi et al., 2014a; Shabnam et al., 2016). Recently, Shabnam et al. (2016) demonstrated the potential of photosynthetic electron transport to reduce Ag^+^ to Ag^0^ and to generate Ag^0^-NPs. They also specifically showed that O_2_ released as a byproduct during photosynthetic electron transport promotes oxidation of Ag^0^ and/or Ag^0^-NPs to generate Ag_2_O-NPs. Therefore, we believe that O_2_ released by light harvesting photosynthetic machinery of leaves promotes oxidation of Ag^0^ and/or Ag^0^-NPs to generate Ag_2_O-NPs.

As evident from the Supplementary Figure 1, only one side of floating leaves is in immediate contact with test solution and further, overall size (i.e., surface area) of submerged leaves is nearly double of floating leaves (Shabnam and Pardha-Saradhi, 2016). In spite of such a vast variation in the surface area in contact with the test solution, the color intensity of the AgNO_3_ solutions incubated with floating leaves was higher than the ones incubated with submerged leaves. This reveals superior potential of floating leaves to generate Ag-NPs compared to submerged leaves. Superior potential of floating leaves to generate Ag-NPs might be due to their superior photosynthetic photochemical reactions (Shabnam et al., 2015; Shabnam and Pardha-Saradhi, 2016).

Inductive coupled plasma analysis revealed that submerged leaves exposed to Ag^+^ possessed 3–4 fold higher levels of silver than floating leaves, which discloses that the uptake of Ag by the former leaves is significantly higher than the latter leaves. Higher levels of Ag in submerged leaves can be accounted to (i) larger surface area (as detailed above) available for uptake of Ag; and (ii) lower potential to reduce Ag^+^ and generate Ag-NPs, compared to floating leaves. We have recently demonstrated that the uptake of Ag by plants of *Spirodela polyrhiza* in the ionic state is 3–4 times higher than in the NP state (Shabnam et al., 2017). Therefore, we believe that superior potential to efficiently reduce Ag^+^ and generate Ag-NPs is one of the mechanisms acquired by floating leaves to curb the uptake of Ag.

### Floating Leaves Possess Superior Potential to Protect Photosynthetic Machinery Against Ag^+^-Toxicity Than Submerged Leaves

Photosystem II plays a vital role in photochemical reactions. Accordingly, overall photosynthetic capacity of plants often relies on PS II activity (Shabnam et al., 2015). Therefore, during the present investigations, we evaluated the impact of Ag^+^ on PS II efficiency. *F*_v_/*F*_m_ is a commonly used parameter to determine Photosystem II efficiency of plants (Strasser et al., 1995; Stirbet and Govindjee, 2012). PS II efficiency, measured in terms of *F*_v_/*F*_m_ as well as through Chl *a* fluorescence kinetics, was significantly higher in floating leaves compared to submerged leaves, just as in our earlier findings (Shabnam et al., 2015).

In this paper, we found that Ag^+^ caused a significant decline in PS II efficiency of both floating and submerged leaves of longleaf pondweed. Ag^+^-induced decline in the quantum yield of PS II activity has also been reported in submerged *P. crispus*, as well as in free floating *S. polyrhiza* (Xu et al., 2010; Jiang et al., 2012; Shabnam et al., 2017). However, Ag^+^-induced suppression in PS II efficiency was significantly lower in the floating leaves compared to that in the submerged leaves. These findings unequivocally demonstrate the prevalence of superior mechanism(s) in floating leaves to counter Ag^+^.

As mentioned earlier, all oxygen-evolving organisms show polyphasic Chl *a* fluorescence transients with distinct O-J, J-I, and I-P photochemical phases (**Figure 3**). While O-J rise (0.05–2 ms) involves the reduction of Q_A_ to Q_A_^-^, the J-I rise (2–30 ms) denotes reduction of PQ pool and the I-P rise (30–300 ms) implies reduction of the acceptor side of PS I (Strasser et al., 1995; Stirbet and Govindjee, 2012; Hamdani et al., 2015; Shabnam et al., 2015, 2017). The OJIP transient kinetics are highly sensitive to various stresses including heavy metal stress (Appenroth et al., 2001; Oukarroum et al., 2012; Shabnam et al., 2015, 2017). During this study, we observed a drastic negative impact of Ag^+^ on the OJIP transients in longleaf pondweed leaves, even at a concentration of 5 μM. Negative impact of Ag^+^ on the OJIP transients was significantly higher in the submerged leaves. As shown in **Figure 3**, while floating leaves retain polyphasic nature of the OJIP transients, the submerged leaves showed a complete loss in the polyphasic nature of this transient on exposure to 5 μM Ag^+^. Severe loss in fluorescence intensity or polyphasic nature of OJIP transients has also been observed in several algae and plants exposed to heavy metals (Appenroth et al., 2001; Oukarroum et al., 2012; Wang et al., 2014; Shabnam et al., 2017). There are reports of inhibition of the oxygen evolving complex (OEC) by metal ions such as Cd and Cr (see e.g., Atal et al., 1991). Severe negative impact of Ag^+^ on the OJIP transients during this study suggests that Ag^+^ could be inhibiting the OEC of PSII as well as the flow of electrons from Q_A_^-^ to the electron acceptor side of PS I (via the PQ-pool), in both floating and submerged leaves of longleaf pondweed. Higher Ag content in submerged leaves could be responsible for a significantly higher decline in their photosynthetic efficiency, compared to that of floating leaves.

As noted earlier, Chl *a* fluorescence kinetics can be affected by such factors like Chl content. A 24-h Ag^+^ treatment caused a decline in the levels of Chl *a* and Chl *b* in a concentration-dependent manner, in both floating and submerged leaves (**Table 2**). However, the decline in the levels of both Chl *a* and Chl *b* were significantly lower in the floating leaves. Our present findings clearly demonstrate that floating leaves are better equipped to protect their photosynthetic machinery against Ag^+^-toxicity than submerged leaves. Superior potential of the floating leaves to withstand Ag^+^ induced suppression of photosynthetic efficiency could be due to (i) higher potential to reduce Ag^+^ to Ag-NPs at the surface, and (ii) restricted uptake of Ag compared to submerged leaves.

In spite of having similar carboxylase activity of Rubisco, the control submerged leaves possess lesser carbon skeletons compared to the control floating leaves. Higher carbon skeletons in floating leaves is due to significantly higher and efficient light harvesting photochemical reactions (pivotal for the generation of assimilatory power essential for CO_2_ fixation and the synthesis of various carbon skeletons). Lower decline in carboxylase activity of Rubisco in floating leaves revealed that floating leaves are better equipped to protect the carboxylase activity of Rubisco than submerged leaves.

Decline in the carboxylase activity of Rubisco has been reported earlier in terrestrial plants, such as *Phaseolus vulgaris, Zea mays, Oryza sativa*, and *Citrus grandis*, exposed to Zn, Cd, and Mn (Van Assche and Clijsters, 1986; Krantev et al., 2008; Li et al., 2010; Wang et al., 2014) and aquatic plants, such as *Salvinia natans* and *Ceratopteris pteridoides*, exposed to Cr and Cd (Dhir et al., 2008; Deng et al., 2014). Ag^+^ induced decline in PS II efficiency and carboxylase activity of Rubisco of these plants may be due to (i) ROS induced inactivation (Sharma and Dietz, 2009; Foyer and Noctor, 2016), (ii) enhanced proteolytic activity (Hajduch et al., 2001; Gajewska et al., 2013), (iii) interference with enzyme’s structure by substitution of native ions (Van Assche and Clijsters, 1986) and/or interaction with SH groups (e.g., by Cu and Cd) (Stiborova, 1998; Stiborova et al., 1998), and (iv) impaired protein biosynthesis (Kremer and Markham, 1982). A simple comparison of the impact of Ag^+^ on PSII efficiency with carboxylase activity of Rubisco reveals that the light harvesting photochemical reactions are more sensitive to Ag^+^ than carbon fixation reactions.

### Superior Antioxidant System of Floating Leaves Counters Ag^+^-Toxicity

In general, heavy metals promote generation of ROS by interfering with electron transport and redox reactions (Prasad et al., 1999; Hall, 2002; Metwally et al., 2003; Sharma and Dietz, 2009; Pardha-Saradhi et al., 2014b). Accordingly, plants have evolved antioxidant systems, both non-enzymatic and enzymatic, to counter oxidative stress (Prasad et al., 1999; Shabnam and Pardha-Saradhi, 2016). Amongst the non-enzymatic antioxidants, ascorbate, phenolics and thiols play important roles in scavenging ROS and/or chelating heavy metals (Sakihama et al., 2002; Shabnam et al., 2014; Shabnam and Pardha-Saradhi, 2016). Plants exposed to heavy metal ions, such as Cd, Pb, and Zn, show enhanced levels of non-enzymatic antioxidants (Prasad et al., 1999; Oncel et al., 2000; Mishra et al., 2006). On the contrary, we observed a decline in the levels of all the non-enzymatic antioxidants in both floating and submerged leaves exposed to Ag^+^ (**Figure 6**). Significantly lower Ag^+^-induced decline in the content of phenolics in floating leaves could be one of the factors contributing to their superior potential to withstand Ag^+^-stress. Posmyk et al. (2009) also reported a decline in the phenolic content in red cabbage under Cu stress. A significant decline in the levels of ascorbate and/or thiols/GSH has been reported in studies with (i) submerged *P. crispus* plants exposed to Ag^+^ (Xu et al., 2010); (ii) pigeon pea seedlings exposed to Ni^2+^ and Zn^2+^ (Rao and Sresty, 2000); (iii) roots of soybean exposed to Cd^2+^ (Balestrasse et al., 2001); and (iv) roots and shoots of maize exposed to Cd^2+^ (Tukendorf and Rauser, 1990; Meuwly and Rauser, 1992). Ag^+^ has a strong affinity for thiols (Rao and Sresty, 2000; Blaske et al., 2013) like other heavy metals. A positive correlation has been established between the depletion of thiol content and the amount of metal ions (Cu^2+^, Zn^2+^) accumulated by plants (Tripathi et al., 2006). Thus, a decline in the levels of thiols, shown in this study, could be due to the binding of thiols to Ag^+^. In general, decrease in the levels of non-enzymatic antioxidants could be due to (i) enhanced catabolic degradation, (ii) alteration in their structure via chelation or reduction of metal ions, and/or (iii) decreased biosynthesis.

Significantly higher activities of antioxidant enzymes in floating leaves impart superior potential to counter oxidative damage compared to submerged leaves. Ag^+^ induced a significant decline in activity of SOD and catalase in both floating and submerged leaves; the decline was significantly higher in the latter (**Figure 7A**). Contrary to the decreased activity of SOD and catalase, the activity of GPX increased by 2–2.5-fold in the submerged leaves exposed to Ag^+^ (**Figure 7C**). However, the floating leaves showed a decrease in GPX activity on exposure to Ag^+^. In contrast to the decline in the activities of SOD and catalase, and an increase in GPX activity recorded in the submerged leaves of longleaf pondweed (present study), Xu et al. (2010) noted increase in activities of SOD and catalase, and a decline in the activity of GPX in submerged *P. crispus*, exposed to Ag^+^. *Salvinia natans* exposed to Cr-rich water, however, showed an increase in GPX activity, without any significant alteration in CAT activity (Dhir et al., 2009). Interestingly, Shah et al. (2001) also noted enhancement of GPX activity accompanied with a decline in CAT activity in rice exposed to cadmium. Peroxidases play a significant role in the synthesis of lignin, which is impermeable to metal ions (Hegedüs et al., 2001; Parrotta et al., 2015). Therefore, we believe that an increased GPX activity in submerged leaves might be a strategy to restrict the uptake of Ag^+^.

Silver ions also suppressed activities of enzymes of the ascorbate glutathione cycle in both types of the leaves. Ag^+^ induced decline in the activities of antioxidant enzymes in both floating and submerged leaves, except GPX in the submerged leaves, which is in agreement with those measured in the roots of soybean and poplar exposed to Cd^2+^ (Balestrasse et al., 2001; Schutzendubel et al., 2002). A similar decline in the activities of several antioxidant enzymes was observed in cotyledons and leaves of sunflower seedlings under Cd^2+^, Fe^2+^, and Cu^2+^ stress (Gallego et al., 1996a,b). In addition, the potential of Ag^+^ to displace native metal cations from their usual binding sites in enzymes has been reported (Ghandour et al., 1988). Ag^+^ induced decline in the activity of the antioxidant enzymes might be due to the effect of Ag^+^ on expression of the relevant genes at the transcriptional/translational level by binding with DNA/RNA. Further, this effect might be at the post-translational level. The binding of Ag^+^ to SH and other active groups might alter 3-D structure of these antioxidant enzymes affecting the catalytic/active site(s) vital for their activities (Ghandour et al., 1988).

**FIGURE 9:**
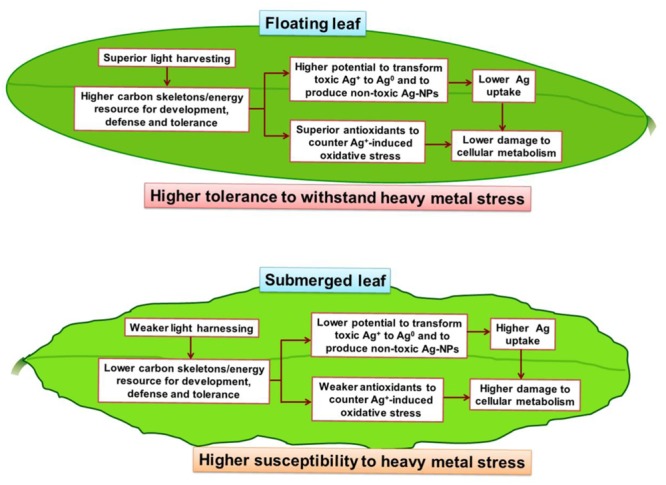
A hypothetical model depicting the key metabolic events in floating and submerged leaves of longleaf pondweed (*P. nodosus*) influenced by Ag^+^-stress. Note that the superior photosynthesis enables floating leaf to be better equipped with carbon skeletons and energy resources required to counter heavy metal stress than submerged leaves.

A summary of the differential impacts of Ag^+^ on the floating and the submerged leaves of longleaf pondweed is presented in a hypothetical model (**Figure 9**). Superior photosynthesis in floating leaves leads to production of more carbon skeletons and energy resources compared to that in submerged leaves. Accordingly, floating leaves are better equipped to counter/tolerate stress imposed by heavy metals, such as Ag^+^. This includes (i) a superior capacity to biotransform toxic ionic state of heavy metals (such as Ag^+^) into less/non-toxic NPs (such as Ag^0^/Ag_2_O-NPs); and (ii) a better capacity to counter oxidative stress through a superior antioxidant system. In addition, significantly higher levels of Ag accumulated in submerged leaves would also directly interfere with their cellular metabolism.

## Conclusion

In this paper, we have demonstrated for the first time that the floating leaves of longleaf pondweed possess a significantly higher potential to withstand Ag^+^-toxicity compared to that in the submerged leaves due to (i) superior photosynthetic machinery and an antioxidant system, (ii) superior potential to reduce Ag^+^ to Ag^0^ and generate Ag-NPs (Ag^0^/Ag_2_O-NPs) on their surface; and (iii) superior potential to restrict uptake of Ag. Our findings suggest that any effort made to increase the proportion of floating leaves to the submerged leaves in longleaf pondweed would be beneficial for apt detoxification of water bodies contaminated with heavy metal ions.

## Author Contributions

The conception or design of the work: PP-S, NS, and HK. The acquisition, analysis: NS, PS, PP-S, and HK. Interpretation of data for the work: NS, PS, PP-S, HK, and G. Drafting the work: NS, PS, and PP-S. Revising it critically for important intellectual content: NS, PS, PP-S, HK, and G. Final approval of the version to be published: NS, PS, PP-S, HK, and G. Agreement to be accountable for all aspects of the work in ensuring that questions related to the accuracy or integrity of any part of the work are appropriately investigated and resolved: NS, PS, PP-S, HK, and G.

## Conflict of Interest Statement

The authors declare that the research was conducted in the absence of any commercial or financial relationships that could be construed as a potential conflict of interest.

## Abbreviations

Chl: chlorophyll
DHA: dehydroascorbate
DTNB: 5,5′-dithiobis-(2-nitrobenzoic acid)
DTT: dithiothreitol
EDTA: ethylene diamine tetra acetic acid
EDX: energy dispersive X-ray
fcc: face cubic centered
*F*_o_: minimum chlorophyll *a* fluorescence
*F*_m_: maximum chlorophyll *a* fluorescence
*F*_v_: variable chlorophyll *a* fluorescence (*F*_m_ -*F*_o_)
GAE: gallic acid equivalent (s)
GPX: guaiacol peroxidase
GR: glutathione reductase
GSH: reduced glutathione
MDA: malondialdehyde
MDHAR: monodehydroascorbate reductase
NAD(P)^+^: oxidized nicotinamide adenine dinucleotide (phosphate)
NAD(P)H: reduced nicotinamide adenine dinucleotide (phosphate)
NEM: *N*-ethylmaleimide
NP: nanoparticle
PS: photosystem
PVP: polyvinylpyrrolidone
PXRD: powder X-ray diffraction
ROS: reactive oxygen species
Rubisco: ribulose 1,5-biphosphate carboxylase/oxygenase
SAED: selected area electron diffraction pattern
SOD: superoxide dismutase
TCA: trichloroacetic acid
TEM: transmission electron microscope
Tris: tris-(hydroxymethyl)-aminomethane
