# Supplementary figures and images for: Differential Response of Floating and Submerged Leaves of Longleaf Pondweed to Silver Ions
Source: Front Plant Sci. 2017 Jun 21;8:1052. doi: 10.3389/fpls.2017.01052 (PMC5478881; doi:10.3389/fpls.2017.01052)

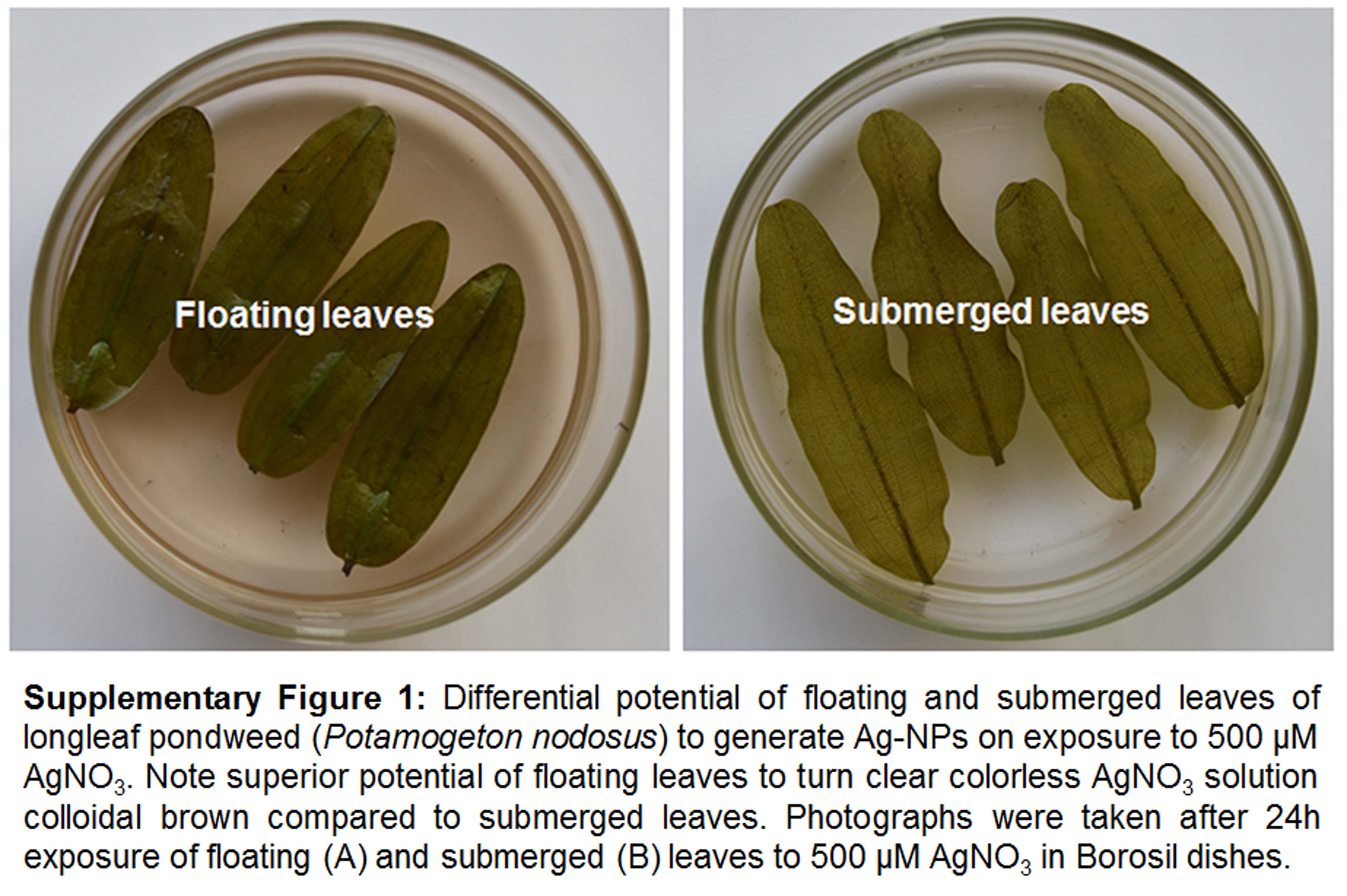

Supplement: Supplementary file 1 [file Image_1.jpg]
